# GC bias lead to increased small amino acids and random coils of proteins in cold-water fishes

**DOI:** 10.1186/s12864-018-4684-z

**Published:** 2018-05-02

**Authors:** Dongsheng Zhang, Peng Hu, Taigang Liu, Jian Wang, Shouwen Jiang, Qianghua Xu, Liangbiao Chen

**Affiliations:** 1 Key Laboratory of Exploration and Utilization of Aquatic Genetic Resources, Shanghai Ocean University, Ministry of Education, National Demonstration Center for Experimental Fisheries Science Education (Shanghai Ocean University), Shanghai, People’s Republic of China; 2 Department of Genetics, University of Pennsylvania, Philadelphia, USA; 3 College of Informatics, Shanghai Ocean University, Shanghai, People’s Republic of China; 4 College of Marine Sciences, Shanghai Ocean University, Shanghai, People’s Republic of China

**Keywords:** Cold adaptation, Substitution bias, Cold-water, Antarctic, Genome, Fish

## Abstract

**Background:**

Temperature adaptation of biological molecules is fundamental in evolutionary studies but remains unsolved. Fishes living in cold water are adapted to low temperatures through adaptive modification of their biological molecules, which enables their functioning in extreme cold. To study nucleotide and amino acid preference in cold-water fishes, we investigated the substitution asymmetry of codons and amino acids in protein-coding DNA sequences between cold-water fishes and tropical fishes., The former includes two Antarctic fishes, *Dissostichus mawsoni* (Antarctic toothfish)*, Gymnodraco acuticeps* (Antarctic dragonfish)*,* and two temperate fishes*, Gadus morhua* (Atlantic cod) and *Gasterosteus aculeatus* (stickleback)*,* and the latter includes three tropical fishes, including *Danio rerio* (zebrafish), *Oreochromis niloticus* (Nile tilapia) and *Xiphophorus maculatus* (Platyfish).

**Results:**

Cold-water fishes showed preference for Guanines and cytosines (GCs) in both synonymous and nonsynonymous codon substitution when compared with tropical fishes. Amino acids coded by GC-rich codons are favored in the temperate fishes, while those coded by AT-rich codons are disfavored. Similar trends were discovered in Antarctic fishes but were statistically weaker. The preference of GC rich codons in nonsynonymous substitution tends to increase ratio of small amino acid in proteins, which was demonstrated by biased small amino acid substitutions in the cold-water species when compared with the tropical species, especially in the temperate species. Prediction and comparison of secondary structure of the proteomes showed that frequency of random coils are significantly larger in the cold-water fish proteomes than those of the tropical fishes.

**Conclusions:**

Our results suggested that natural selection in cold temperature might favor biased GC content in the coding DNA sequences, which lead to increased frequency of small amino acids and consequently increased random coils in the proteomes of cold-water fishes.

**Electronic supplementary material:**

The online version of this article (10.1186/s12864-018-4684-z) contains supplementary material, which is available to authorized users.

## Background

Temperature adaptation is a fundamental issue in evolutionary biology. Genomic adaptation, especially modification of sequence and structure of the biological molecules, including DNAs and proteins, are necessary for improving their functionality under extreme temperature. Genomic GC content and its relationship with temperature adaptation have been studied for a long time but are still under debate. Comparison of cold- and warm-blooded vertebrate genomes showed an increment of GC heterogeneity in warm-blooded species [1–5]. On the other hand, both polar and temperate fishes showed a GC level significantly higher than that of tropical and sub-tropical fishes [6–8]. Studies on prokaryotes indicated that hyperthermal species have higher GC contents than mesothermal species [9–11]. But a generalized relationship was questioned by other studies [12, 13]. Based on these observations, different proposals have been raised to explain how GC content could affect environmental adaptation. For example, as GC content reflects the degree of hydrogen bonding in DNA double helices, an early study proposed that higher GC contents lead to an increased thermal stability in DNA chains under high temperature [14]. Recently, some hypotheses focused on possible regulatory mechanisms of GC-rich regions. Vinogradov proposed that increase in GC content may lower the energy barrier of the B-Z conformation transition in DNA isochore and facilitate response to environmental stress [15]; Galtier et al. hypothesized that increased GC could promote the genome evolution and adaptation to the environment [16], while a recent study indicated mRNA level was positively correlated with GC content in the third nucleotide site of codons, indicating an important role of GC content in regulating gene expression level [17]. For coding DNA sequences (CDS), the genetic code defines how DNA sequences are translated into amino acids. GC bias in CDS has been shown to lead to an overall bias in the amino acid composition of proteins in some comparative genomic studies [18–21]. But these studies did not address whether ambient temperature could shape the GC content in DNA and amino acid preferences of proteins.

Proteins are sensitive to temperature change. Rates of most biochemical reactions are 2–3 folds slower for every 10 °C decreased in temperature because of the decreased kinetic energy. Many proteins are structurally modified to maintain proper levels of functionality under extreme temperature conditions. For example, in a comparative study on 16 protein families, Gromiha et al. found that the increase in shape (location of branch point in side chain) of amino acid increases the themostability of proteins [22]. Studies on protein structure and activities of muscle-specific A4-lactate dehydrogenases (A4-LDHs) in polar fishes showed that these proteins have increased catalytic rates when comparing with their orthologs from fishes living at higher temperatures, which allows them to maintain similar substrate affinity at the freezing temperatures [23]. Further studies showed that amino acid substitutions in this enzyme leads to local secondary structure change, which increases the flexibility of the enzyme and lowers the energy barrier of the reaction in Antarctic fishes [23, 24]. Other comparative studies on microtubule [25], malate dehydrogenase [26], RTX lipase [27] and phosphoglycerate kinase [28] also showed that proteins from polar species demonstrated higher structural flexibility. Comparative genomic studies on prokaryotes revealed that psychrophiles prefer amino acids with tiny/small or neutral side chains, which contribute to higher flexibility by having more coils and less helices in the secondary structure [29–31]. Despite many studies on temperature adaptation of individual enzymes, genomic studies on amino acid bias in cold adaptation in vertebrates are still scarce.

Being ectothermal vertebrates, fishes have developed comprehensive mechanisms to adapt to broad ranges of thermal conditions and colonized almost all of the aquatic habitats on the Earth, from tropical regions to the Polar Regions. Freezing water temperatures in the Polar Regions pose a challenge for the survival of fishes. The Arctic regions vary among different locations. Furthermore, seasonal shifts and daily fluctuations occur in the lower Arctic and subarctic latitudes [32]. Temperate fishes living in these regions, including cod (*Gadus morhua*) and stickleback (*Gasterosteus aculeatus*), can tolerate the temperature fluctuation [33, 34]. In contrast, water temperatures of the Antarctic region are highly cold stable, ranging from − 1.9 °C in the high Antarctic latitudes to + 3 °C in the low Antarctic latitudes [35, 36]. At high Antarctic latitudes, temperatures seldom deviate from freezing throughout the life span of the local fish species [37, 38]. Antarctic notothenioids adapted to such extreme cold conditions and form a stenothermal fauna that has a poor capacity to tolerate elevated body temperature [39]. Various molecular responses have been described in cold adaptation in Antarctic fish, such as acquisition of antifreeze glycoproteins (AFGP) [40] and functional diversification of Zona Pellucida proteins [41], loss of heat shock response [42] and remodeling of the haematopoietic programs [43]. The diversity of the thermal adaptation makes fishes good models for studying molecular mechanisms of temperature adaptation.

As more and more fish genomic data are available, it is now possible to investigate the effects of ambient temperature on codon and amino acid evolution in fish genomes. Substitutional asymmetry based on the homologous sequence alignment of species pairs have proven reliable in genome evolution studies [9, 44–46]. To investigate how cold temperature may influence evolution of codon and amino acid preferences in fishes, we compared protein coding sequences between cold-water fishes and tropical fishes using substitutional asymmetry analysis. *Gasterosteus aculeatus* (three-spin stickleback) from Bear Paw Lake in Alaska, and *Gadus morhua* (Atlantic cod) were taken as temperate fish models in this study. *Dissostichus mawsoni* (Antarctic toothfish) and *Gymnodraco acuticeps* (Antarctic dragonfish) were taken as Antarctic stenothermal fish models. Three tropical fishes were taken as references in this study, including *Danio rerio* (zebrafish), *Oreochromis niloticus* (tilapia) and *Xiphophorus maculatus* (Platyfish). We found increased GC content in codons in cold-adapted fishes, which leads to increased ratios of small amino acids and random coils in proteomes of the polar fishes.

## Methods

### Sequencing and data collection

Two Antarctic notothenioids *D. mawsoni* and Two *G. acuticeps* were captured from McMurdo Sound and Prydz Bay near the China Zhongshan Station, respectively. Tissues were dissected from anesthetized specimens and kept frozen at − 80 °C until use. For sequencing the transcriptomes of *D. mawsoni* and *G. acuticeps*, mixed tissue samples were homogenized and the mRNA was extracted using Oligotex mRNA Isolation Kit (Qiagen, CA, USA). The quantity of total RNA was determined using a Qubit fluorometer (Life Technologies). The quality of RNA was assessed by measuring RINs using Bioanalyzer Chip RNA 7500 series II (Agilent). 3μg of total RNA from each sample was used to prepare mRNA-Seq library with TruSeq™ RNA Sample Prep Kit (Illumina), following the manufacturer’s instructions. Library quality control was performed with a bioanalyzer Chip DNA High Sensitive (Agilent). Each library had an insert size of 300-400 bps, and 2X 100 bps paired-end sequences were generated using Hiseq 1500 (Illumina). After removing adaptor sequences and filtering out sequences with unknown nucleotides or low quality (quality scores< 20), De Novo assembly of the reads was performed using Trinity with default settings. The resultant data were processed with CD-HIT-EST to eliminate redundancy.

The protein sequences and the corresponding coding DNA sequences (CDS) of the following fish genomes were downloaded from ensembl database: *G. morhua* (gadMor1.72), *G. aculeatus* (BROADS1.72), *D.rerio* (Zv9.72), *X.maculatus* (Xipmac4.4.2.72) and *O.niloticus* (Orenil1.0.72). Sequences with unidentified nucleotides or amino acids were excluded for further analysis. Information about Geographical distribution and habitat temperature of fishes were retrieved from www.fishbase.org.

### Phylogenetic tree reconstruction

Mitogenome sequences of fishes investigated in this study were downloaded from NCBI database, the 13 protein-coding genes were aligned using the program CLUSTAL, MEGA7 [45] was used to choose the best nucleotide substitution model. According to the lowest BIC (Bayesian Information Criterion) scores and the number of parameters, The GTR + G + I was selected for the concatenated nucleotide sequence alignment of the 13 protein-coding genes. Maximum likelihood method was applied for phylogenetic tree reconstruction using MEGA7.

### Identification orthologous gene pairs

To investigate substitutional asymmetry of each cold-water fish against tropical fishes, we identified putative orthologous gene pairs between cold-water fish and each of the tropical fishes using BLASTP bidrectional best hit (BBH) approach with a cutoff e-value of 10^− 5^. The pairwise alignments of putative orthologous sequences, which were retrieved from BBH hits with the length more than 20 amino acids and similarity more than 50%, were further analyzed for substitutional asymmetry in codon and amino acid usage. Considering substitution, we refer only to the fact that we analyzed the nucleotide and amino acid changes between the putative orthologous sites of the putative orthologous sequences.

### Evaluation of GC bias in polar fishes

Pairwise alignments of the CDS were achieved by reverting the protein alignments to corresponding CDS alignments using program pal2nal.pl [47]. Nucleotide substitutions were classified as nonsynonymous substitutions and synonymous substitutions, based on whether or not a substitution results in an amino acid change. For both situation, the counts that G/C in cold-water fish are substituted by A/T in tropical fish (s1) and the counts that A/T in cold-water fish are substituted by G/C in tropical fish (s2) were calculated for each species pair, and the ratio between these two counts (s1/s2) were taken as GC bias ratio in cold-water fish. If the bias ratio is above 1, it would indicate that GC is preferred by the polar fish in the species pair.

Relative Synonymous Codon Usage (RSCU) of the CDS dataset for each species was calculated using the program CodonW 1.4.2 (http:// codonw.sourceforge.net/) [48] to further investigate codon usage bias for each amino acid. To evaluate GC bias in codon usage, we summed the normalized frequencies of GC-rich codons and GC-poor codons respectively, which code for the same amino acid, and the bias was calculated by dividing the sum of GC-rich codons by that of GC-poor codons.

### Calculating amino acid bias ratios in polar fishes

Amino acid substitutions were classified by the two amino acids involved in substitution (20 amino acids result in 20*19 = 380 amino acid substitution types in total). To investigate amino acid bias in cold-water fishes, we calculated bias ratios for each amino acid following the same idea as GC bias ratio calculation. For each species pair, we calculated the count that an amino acid in the cold-water fish is substituted by other amino acids in the tropical fish, and the count that the amino acid in the tropical fish is substituted by other amino acids in the cold-water fish. The ratio between the two counts is defined as the bias ratio of the amino acid in the polar fish.

The same strategy was also applied to study the properties of amino acids in cold-water fishes. We collected some related properties from the amino acid index database AAindex [49], including molecular weight, size, graph, polarity and hydrophobicity (Additional file 1), and analyzed their bias in the substitution. For example, to study bias of molecular weight in cold-water fishes, we calculated the counts that an amino acid with a smaller molecular weight in the polar fish is substituted by an amino acid with larger molecular weight in the tropical fish and vice versa, and calculated ratios between the two frequencies as the molecular weight bias ratios.

### Secondary structure prediction

To investigate secondary structure preference in different fishes, we predicted the secondary structure for protein sequences using PSIPRED with default parameters [50]. The secondary structure elements were classified as helices, strands and random coils by this software. The predicted secondary structure element sequences were aligned based on their corresponding protein sequence alignments. As PSIPRED prediction accuracy is about 70–80%, for each pairwise alignment, we kept the sites with prediction confidence scores above 5 for both putative orthologous sites to filter out sites with unreliable prediction. Further analysis of the secondary structure bias followed the same procedure as for amino acid substitution analysis.

#### Statistics

All the statistical tests were performed using R and Excel. When testing whether substitution is biased or unbiased for a group of data, one sample t-test was carried out to examine if the bias ratio has statistically significant difference from 1, two sample t-test or paired t-tests were carried out when comparing bias ratios between two groups when applicable. Furthermore, to test substitution asymmetry for each species pair, we used tests of goodness-of-fit under a chi-square distribution to compare the numbers of substitutions in both directions. Linear regression analysis was applied to investigate the relationship between GC bias ratios and averaged GC content for codons coding for the same amino acid. Spearman’s rank correlation coefficients were calculated to examine the relationship between GC content of codons and their bias ratios for nonsynonymous substitutions in polar fishes.

## Results

### RNA sequencing, data retrieval and sequence alignment of the homologous fragements

We sequenced mRNA of *D. mawsoni* and *G. acuticeps* using Hiseq 1500 (Illumina). The total numbers of reads were 29,275,193 and 58,422,615 for *D. mawsoni* and *G. acuticeps*. There were 112,705 contigs assembled, with average contig size of 1030 bp for *D. mawsoni*; for *G. acuticeps*, there were 94,141 contigs were assembled, with average contig size of 745 bp.

**Fig. 1:**
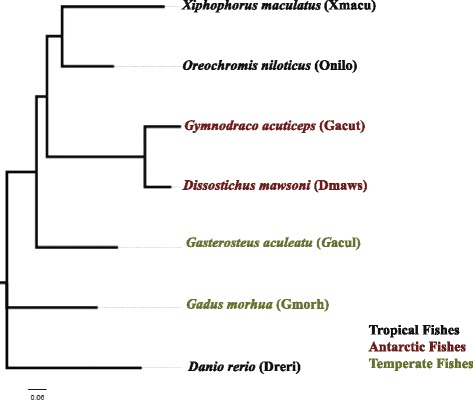
Fishes investigated in this study

The contigs of *D. mawsoni* and *G. acuticeps*, together with the sequences downloaded from Ensembl website, were analyzed to investigate substitutional asymmetry between polar fishes and tropical fishes. Information of the fishes covered in this study is summarized in Fig. 1 and Additional file 2. The sizes of alignments showed that transcriptome sequencing of the two Antarctic fishes provided us data size comparable to other fish genomes (Additional file 2).

### GC bias in polar fishes is linked to amino acid substitution bias

**Fig. 2:**
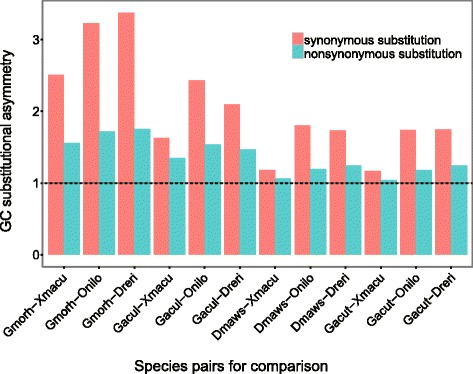
GC to AT bias ratios between the polar fish and the tropical fish for each species pair. All GC to AT bias ratios are above 1, indicating that GC is preferred in polar fishes in both synonymous and nonsynonymous substitutions

To study GC bias in cold-water fishes, we calculated GC bias ratios between the cold-water fish and the tropical fish for each species pair. As shown in Fig. 2, all the bias ratios are above 1, indicating that GC is preferred in all cold-water fishes for both synonymous and non-synonymous substitution when compared with tropical fishes, which is consistent with previous comparative studies on fish genomes [6–8]. To compare the numbers of substitutions in both directions, we also applied chi-squared test, which also showed that GCs are increased in the cold-water fish for all species pairs (Additional file 3: Tables S3 and S4). Figure 2 also showed that synonymous substitutions are more biased than non-synonymous ones (one-tailed pairwise t-test, df = 5, *P*-value< 0.01). Furthermore, Bias ratios in temperate fishes are more deviate than in Antarctic fishes for both synonymous and non-synonymous substitutions (one-tailed t-tests, df = 5, *P*-values< 0.01).

We further examined bias ratios for individual codons. As shown in Additional file 4: Table S5, for synonymous substitutions, the bias ratios of codons displayed a binomial model: if the third position of the codon is G/C, the bias ratio for cold-water fishes is above 1 in most cases; otherwise, it is mostly below 1. These results indicate that cold-water fishes prefer G/C as the third nucleotide of the genetic codes. The only exception is TTG, which is disfavored in all cold-water fishes. Consistent with these results, chi-squared tests of the distributions also showed that GC-rich codons are preferred in cold-water fishes (Additional file 5: Table S7 and S8). We also investigated codon usage bias by calculating Relative Synonymous Codon Usage (RSCU) of the whole CDS dataset for each species. As shown in Additional file 6: Table S9, when compared with tropical fishes, cold-water fishes prefer GC-rich codons to AT-rich codons for synonymous codons in most cases.

**Fig. 3:**
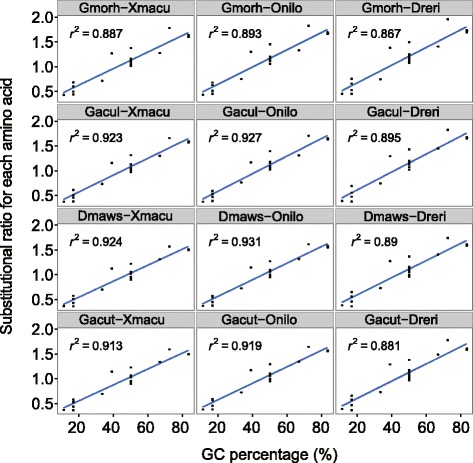
Relationship between GC to AT bias ratios and average GC percentages of codons for each amino acid

To investigate the GC bias in nonsynonymous substitutions, we classified the standard genetic codes into four groups (0 to 3) based on the number of GCs present in the genetic codes, as shown in Additional file 4: Table S6, and tested the correlations between the GC content and their GC bias ratios in nonsynonymous substitutions for each species pairs using sperman’s rank correlation coefficient. As shown in Additional file 7: Table S10, the bias ratios are correlated with the GC content in the genetic codes, indicating that GC-rich codons are favored in cold-water fishes. As nonsynonymous substitution will lead to amino acid change, we further investigate how GC bias may affect amino acid bias in cold-water fishes. We plotted the averaged GC content of genetic codes encoding the same amino acid against their GC bias ratios in nonsynonymous substitution for each species pair. As shown in Fig. 3, in terms of amino acids, GC bias ratios are positively correlated with the averaged GC content of the codons in nonsynynomous substitution (the coefficient of determination > 0.86, df = 18 for each species pair), indicating that cold-water fishes favors amino acids that coded by GC rich codons.

#### Amino acid substitution bias

**Fig. 4:**
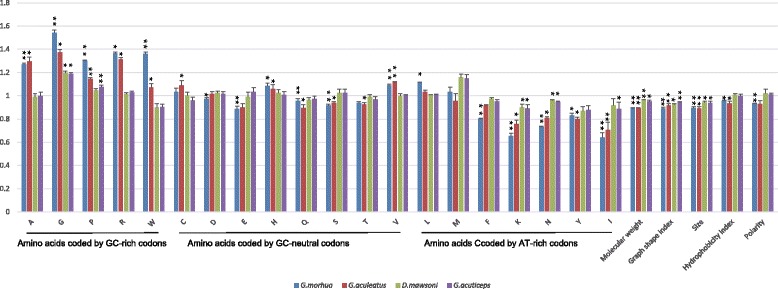
Amino acid substitutional bias in polar fishes. The bias ratios of 20 amino acids and specific properties for polar fishes were calculated and the significance of their difference from 1 were labelled with *: *P*-value< 0.05, **: *P* < 0.01

To investigate the overall preference of the amino acid substitution in fishes, for each cold-water fish, we calculated the bias ratios of each amino acid against the three tropical fishes, and test its deviation from unbiased substitutions (assuming the bias ratio to be 1) using one sample t-test with degree of freedom (df) equal to 2, the results are summarized in Fig. 4. The results suggest that substitutions of most amino acids are biased when comparing the temperate fishes with the tropical fishes. The amino acids that coded by the GC-rich codons, including Glycine (encoded by GGN), Alanine (GCN), Proline (CCN), Arginine (CGN) and Tryptophan (TGG) are favored, while most amino acids that coded by the AT-rich codons, including Isoleucine (ATY), Phenylalanine (TTY) and Glutamine (CAA) and Lysine (AAR) are disfavored in *G. morhua*. Interestingly, though Isoleucine is disfavored, Leucine, who has similar biochemical property to Isoleucine but partially coded by GC-rich codons, is favored in *G. morhua*. Furthermore, small amino acids with molecular weight less than 117 Da are mostly favored except Serine (AGN), while amino acids with medium size range from 119 to 147 Da are mostly disfavored in *G. morhua*. Similar result was also present in *G.aculeatus*. Antarctic fishes showed similar trend but not significant in most cases. Glycine is the only amino acid that is favored in all the cold-water fishes, and Glutamine is the only common one disfavored in all the cold-water fishes. When Chi-squared test is applied to test the distribution of amino acid between the species pairs, we found that Glycine and Procine are the two common amino acids favored in all cold-water fishes, and that Glutamine, Lysine, Asparagine and Tyrosine are commonly disfavored in these fishes (Additional file 8: Table S11).

As many small amino acids are preferred in cold-water fishes, we further investigated the substitution patterns on their properties related to the size. Both chi-squared test and t-test indicated that amino acid distribution are biased in cold-water fishes in terms of molecular weight, residue volumes and graph shape (Fig. 4 and Additional file 8: Table S12).

To investigate the possible connection between these properties of amino acids and GC content of the codons coding them, we calculated the correlation between some related numerical property indices of amino acids and averaged GC percentages for the standard genetic codes encoding them, and found that their properties are negatively correlated with GC content of their codons to some degree (Pearson’s correlation coefficient = − 0.43, − 0.39 and − 0.36, *P*-values = 0.06, 0.09 and 0.12 for “size”, “graph shape index” and “molecular weight”, respectively), indicating that GC-rich codons tend to code for small amino acids.

To further investigate if amino acid size is an adaptive property in cold-water fishes, we also examined substitutional asymmetry for amino acids that differ in size but are coded by codons that are equally GC-rich. The bias ratios are above 1.19 for both Antarctic and temperate fishes, indicating that it is more frequently that amino acids get smaller in substitution in cold-water fishes. Chi-square tests for individual species pairs also confirmed this trend in cold-water species (Additional file 9: Table S13).

### Secondary structure substitution bias

**Fig. 5:**
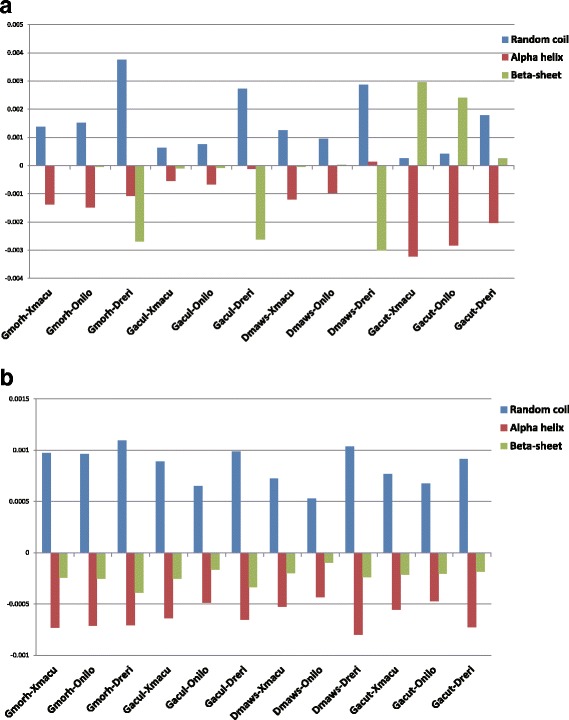
Secondary structure changes for all species pairs. Pane **a** shows the percentages of secondary structure elements net changes in total secondary structure changes for each species pair. Pane **b** shows the effect of molecular weight on secondary structure change. When only the orthologous sites with smaller amino acids in polar fishes than that in tropical fishes are considered, coils are increased and helices are decreased for all species pairs

As amino acid bias may lead to secondary structure change, we investigated the effect of the amino acid substitution preference on the protein secondary structure changes, we predicted the secondary structure of the proteins and investigated secondary structure changes among the pairwise aligned protein sequences. To keep secondary structure prediction reliable, we filtered out the sites with prediction confidence index less than 5, which kept 61.39 ± 1.48% of the original prediction data on average. The most striking secondary structure element change in cold-water fishes is increase of the coils, as shown in Fig. 5a, Additional file 10: Table S14. temperate fishes show significantly more coil increase when compared with Antarctic fishes (6.22 ± 3.27% increase for Arctic fishes and 3.69 ± 2.28% increase for Antarctic fishes, one-tailed t-test, df = 5,*P*-value = 0.076).

Since temperate fishes prefer small amino acids than tropical fishes, we also investigated the effect of molecular weight on secondary structure distribution. As shown in Fig. 5b and Additional file 10: Table S15, when only the orthologous s sites with smaller amino acids in cold-water fishes than that in tropical fishes are considered, all species pairs show increase of coils and decrease of helix and beta-sheets, indicating that smaller amino acids contribute to increase of random coils in cold-water fishes.

## Discussion

DNA and proteins are basic building blocks of life, elucidating how these molecules adapt to temperature is important for understanding mechanisms of evolution. In this study, we investigated preferences of codons and amino acids in cold-water fishes by comparing the putative orthologous gene segments with those of tropical fishes. We found that GC content is increased in protein coding regions in cold-water fishes, which lead to biased amino acid composition in cold-water fishes, including preference of small amino acids. These changes in turn explain the increase of random coils in the secondary structure elements, leading to the increase of structural flexibility of proteins in cold-water fishes.

Codon usage is shaped by many factors. A variety of hypotheses are proposed to explain mechanisms driving codon bias among organisms, some factors are neutral, such as mutational bias, DNA repair mechanisms; while others are selective, including adaptation for optimal translation, and amino acid substitution related to protein functionality. In this study, Increased GC content was found in both Synonymous and non-synonymous substitutions in cold-water fishes when comparing with tropical fishes, which is consistent with previous comparative studies on fish genomes [6–8]. Furthermore, our analysis showed that GC content of the standard genetic codes and size of amino acids are correlated, indicating that GC bias in codons may lead to increase of small amino acids in cold-water fishes. Our amino acid substitution analysis confirmed that small amino acids are preferred in cold-water fishes, and that random coils are increased in these species when comparing with tropical fishes. Tiny and small amino acids tend to form random coils in protein secondary structure and increase the structural flexibility, and they are preferred in cold-adapted proteins [23–28, 51]. Consistent with these observations, our results indicates that small amino acid preference lead to increase of coils in protein secondary structure and cause increase of protein structure flexibility in cold-water fishes.

Although temperate fishes and Antarctic fishes showed some common features in our study, GC content bias and small amino acid content in the two temperate fishes are much more biased than that of Antarctic fishes when both of them are compared with tropical fishes. The difference may result from their adaptation to the different ambient environment. Temperate fishes are exposed to temperature fluctuations, while the Antarctic fishes are highly stenothermal polar fishes. A comparative study on Myoglobin (Mb) showed that Mb from the eurythermal fish mackerel has increased flexibility comparing with its orthologs from stenothermal species [52]. Furthermore, Arctic eurythermal fish *Zoarces viviparus* has higher GC content and small amino acid content than a closely-related Antarctic stenothermal fish *Pachycara brachycephalum* [53], which is consistent with our results. These studies, together with our study, indicates that high GC content leads to increased content of small amino acids and increased protein flexibility in temperate fishes, which may be required for adapting to cold and fluctuating temperature in these regions.

## Conclusions

In summary, our data indicated that protein flexibility is increased in cold-water fishes by having higher GC content in protein coding regions, which may be an important evolutionary mechanism for cold adaptation in cold-water fishes. It will be of great interest to know how temperature exerts pressure on GC bias in DNA, at least in protein coding regions. Many questions need to be addressed in future studies. For example, what genes are mostly affected by GC biased codon substitution? What molecular mechanisms are underlying this kind of substitution? As three of the cold-water species are marine, and sticklebacks are primarily marine or anadromous, while all three tropical species are freshwater, we could not exclude the possibility that salinity may play a role in shaping GC content. Furthermore, many factors may be responsible for the biological molecule preference of a genome, and different strategies may be developed by different organisms to cope with temperature adaptation. As the species pairs are not phylogenetically independent in this study due to availability of genomes of suitable species, which may introduce some bias in our results, more studies are needed to clarify the relationship between biological molecule preference and temperature adaptation.

## Additional files

Additional file 1: **Table S1.** numerical indices of physicochemical and biochemical properties of amino acids. (XLSX 11 kb)

Additional file 2: **Table S2.** Summary of the fishes investigated in this study. (DOCX 14 kb)

Additional file 3: **Table S3.** GC/AT synonymous substitution counts between cold-water and tropical species. **Table S4.** GC/AT nonsynonymous substitution counts between cold-water and tropical species. (XLSX 16 kb)

Additional file 4: **Table S5.** Codon usage bias ratios for synonymous substitutions between codons ending with G/C and those ending with A/T. **Table S6.** Codon usage bias ratios for nonsynonymous substitutions between codons ending with G/C and those ending with A/T. (XLSX 32 kb)

Additional file 5: **Table S7.** Codon substitution for synonymous substitutions between codons ending with G/C and those ending with A/T. **Table S8.** Codon substitution for nonsynonymous substitutions between codons ending with G/C and those ending with A/T. (XLSX 58 kb)

Additional file 6: **Table S9.** Statistics from condonw analysis. (XLSX 22 kb)

Additional file 7: **Table S10.** Sperman’s rank correlation coefficient between codon bias ratio and GC content of codons. (XLSX 12 kb)

Additional file 8: **Table S11.** Amino acid substitution between cold-water and tropical fishes. **Table S12.** Amino acid property changes between cold-water and tropical fishes. (XLS 76 kb)

Additional file 9: **Table S13.** Molecular Weight change of amino acids resulting from substitutions between equally GC-rich codons. (XLSX 11 kb)

Additional file 10: **Table S14.** Secondary structure change between cold-water fishes and tropical fishes. **Table S15.** Secondary structure change between cold-water fishes and tropical fishes when molecular weight is decreased in cold-water fishes. (XLSX 15 kb)

## Abbreviations

CDS: Coding DNA sequences
Dmaws: *Dissostichus mawsoni*
Dreri: *Danio rerio*
Gacul: *Gasterosteus aculeatus*
Gacut: *Gymnodraco acuticeps*
Gmorh: *Gadus morhua*
Mb: Myoglobin
Onilo: *Oreochromis niloticus*
RSCU: Relative Synonymous Codon Usage
Xmacu: *Xiphophorus maculatus*

## References

[1] Bernardi G, Bernardi G (1990). Compositional transitions in the nuclear genomes of cold-blooded vertebrates. J Mol Evol.

[2] Eyre-Walker A, Hurst LD (2001). The evolution of isochores. Nat Rev Genet.

[3] Thiery JP, Macaya G, Bernardi G (1976). An analysis of eukaryotic genomes by density gradient centrifugation. J Mol Biol.

[4] Bernardi G (2007). The neoselectionist theory of genome evolution. Proc Natl Acad Sci U S A.

[5] Costantini M, Cammarano R, Bernardi G (2009). The evolution of isochore patterns in vertebrate genomes. BMC Genomics.

[6] Chaurasia A, Tarallo A, Berna L, Yagi M, Agnisola C, D'Onofrio G (2014). Length and GC content variability of introns among teleostean genomes in the light of the metabolic rate hypothesis. PLoS One.

[7] Costantini M, Auletta F, Bernardi G (2007). Isochore patterns and gene distributions in fish genomes. Genomics.

[8] Uliano E, Chaurasia A, Berna L, Agnisola C, D'Onofrio G (2010). Metabolic rate and genomic GC: what we can learn from teleost fish. Mar Genomics.

[9] Nishio Y, Nakamura Y, Kawarabayasi Y, Usuda Y, Kimura E, Sugimoto S, Matsui K, Yamagishi A, Kikuchi H, Ikeo K (2003). Comparative complete genome sequence analysis of the amino acid replacements responsible for the thermostability of Corynebacterium efficiens. Genome Res.

[10] Musto H, Naya H, Zavala A, Romero H, Alvarez-Valin F, Bernardi G (2004). Correlations between genomic GC levels and optimal growth temperatures in prokaryotes. FEBS Lett.

[11] Galtier N, Lobry JR (1997). Relationships between genomic G+C content, RNA secondary structures, and optimal growth temperature in prokaryotes. J Mol Evol.

[12] Hurst LD, Merchant AR (2001). High guanine-cytosine content is not an adaptation to high temperature: a comparative analysis amongst prokaryotes. Proc Biol Sci.

[13] Musto H, Naya H, Zavala A, Romero H, Alvarez-Valin F, Bernardi G (2006). Genomic GC level, optimal growth temperature, and genome size in prokaryotes. Biochem Biophys Res Commun.

[14] Wada A, Suyama A (1986). Local stability of DNA and RNA secondary structure and its relation to biological functions. Prog Biophys Mol Biol.

[15] Vinogradov AE (2003). DNA helix: the importance of being GC-rich. Nucleic Acids Res.

[16] Galtier N, Piganeau G, Mouchiroud D, Duret L (2001). GC-content evolution in mammalian genomes: the biased gene conversion hypothesis. Genetics.

[17] Chen S, Li K, Cao W, Wang J, Zhao T, Huan Q, Yang YF, Wu S, Qian W: Codon-resolution analysis reveals a direct and context-dependent impact of individual synonymous mutations on mRNA level. Mol Biol Evol 2017:epub ahead of print. 10.1093/molbev/msx229 PMC5850819 28961875

[18] Albu M, Min XJ, Golding GB, Hickey D (2009). Nucleotide substitution bias within the genus Drosophila affects the pattern of proteome evolution. Genome Biol Evol.

[19] Friedman R, Drake JW, Hughes AL (2004). Genome-wide patterns of nucleotide substitution reveal stringent functional constraints on the protein sequences of thermophiles. Genetics.

[20] Paila U, Kondam R, Ranjan A (2008). Genome bias influences amino acid choices: analysis of amino acid substitution and re-compilation of substitution matrices exclusive to an AT-biased genome. Nucleic Acids Res.

[21] Zhou HQ, Ning LW, Zhang HX, Guo FB (2014). Analysis of the relationship between genomic GC content and patterns of base usage, codon usage and amino acid usage in prokaryotes: similar GC content adopts similar compositional frequencies regardless of the phylogenetic lineages. PLoS One.

[22] Gromiha MM, Oobatake M, Sarai A (1999). Important amino acid properties for enhanced thermostability from mesophilic to thermophilic proteins. Biophys Chem.

[23] Fields PA, Somero GN (1998). Hot spots in cold adaptation: localized increases in conformational flexibility in lactate dehydrogenase A4 orthologs of Antarctic notothenioid fishes. Proc Natl Acad Sci U S A.

[24] Fields PA, Houseman DE (2004). Decreases in activation energy and substrate affinity in cold-adapted A4-lactate dehydrogenase: evidence from the Antarctic notothenioid fish Chaenocephalus aceratus. Mol Biol Evol.

[25] Detrich HW (1997). Microtubule assembly in cold-adapted organisms: functional properties and structural adaptations of tubulins from antarctic fishes. Comp Biochem Physiol A Physiol.

[26] Kim SY, Hwang KY, Kim SH, Sung HC, Han YS, Cho Y (1999). Structural basis for cold adaptation. Sequence, biochemical properties, and crystal structure of malate dehydrogenase from a psychrophile Aquaspirillium arcticum. J Biol Chem.

[27] Mohamad Ali MS, Mohd Fuzi SF, Ganasen M, Abdul Rahman RN, Basri M, Salleh AB (2013). Structural adaptation of cold-active RTX lipase from Pseudomonas sp. strain AMS8 revealed via homology and molecular dynamics simulation approaches. Biomed Res Int.

[28] Bentahir M, Feller G, Aittaleb M, Lamotte-Brasseur J, Himri T, Chessa JP, Gerday C (2000). Structural, kinetic, and calorimetric characterization of the cold-active phosphoglycerate kinase from the antarctic Pseudomonas sp. TACII18. J Biol Chem.

[29] Metpally RP, Reddy BV (2009). Comparative proteome analysis of psychrophilic versus mesophilic bacterial species: insights into the molecular basis of cold adaptation of proteins. BMC Genomics.

[30] De Vendittis E, Castellano I, Cotugno R, Ruocco MR, Raimo G, Masullo M (2008). Adaptation of model proteins from cold to hot environments involves continuous and small adjustments of average parameters related to amino acid composition. J Theor Biol.

[31] Saunders NF, Thomas T, Curmi PM, Mattick JS, Kuczek E, Slade R, Davis J, Franzmann PD, Boone D, Rusterholtz K (2003). Mechanisms of thermal adaptation revealed from the genomes of the Antarctic archaea Methanogenium frigidum and Methanococcoides burtonii. Genome Res.

[32] Thomas DN, Fogg GE (2008). The biology of polar regions.

[33] Claireaux G, Webber D, Kerr S, Boutilier R (1995). Physiology and behaviour of free-swimming Atlantic cod (Gadus morhua) facing fluctuating temperature conditions. J Exp Biol.

[34] Guderley H, Leroy PH, Gagne A (2001). Thermal acclimation, growth, and burst swimming of threespine stickleback: enzymatic correlates and influence of photoperiod. Physiol Biochem Zool.

[35] Littlepage JL, Llano GA (1965). Oceanographic investigations in McMurdo sound, Antarctica. Biology of the Antarctic seas II.

[36] DeVries ALS, Steffensen JF, APFJF S (2005). The Arctic and Antarctic polar marine environments. The physiology of polar fishe.

[37] Cziko PA, DeVries AL, Evans CW, Cheng CH (2014). Antifreeze protein-induced superheating of ice inside Antarctic notothenioid fishes inhibits melting during summer warming. Proc Natl Acad Sci U S A.

[38] Hunt BM, Hoefling K, Cheng CHC (2003). Annual warming episodes in seawater temperatures in McMurdo sound in relationship to endogenous ice in notothenioid fish. Antarct Sci.

[39] Peck LS, Morley SA, Clark MS (2010). Poor acclimation capacities in Antarctic marine ectotherms. Mar Biol.

[40] Chen L, DeVries AL, Cheng CH (1997). Evolution of antifreeze glycoprotein gene from a trypsinogen gene in Antarctic notothenioid fish. Proc Natl Acad Sci U S A.

[41] Cao L, Huang Q, Wu Z, Cao DD, Ma Z, Xu Q, Hu P, Fu Y, Shen Y, Chan J (2016). Neofunctionalization of zona pellucida proteins enhances freeze-prevention in the eggs of Antarctic notothenioids. Nat Commun.

[42] Hofmann GE, Lund SG, Place SP, Whitmer AC (2005). Some like it hot, some like it cold: the heat shock response is found in New Zealand but not Antarctic notothenioid fishes. J Exp Mar Biol Ecol.

[43] Xu Q, Cai C, Hu X, Liu Y, Guo Y, Hu P, Chen Z, Peng S, Zhang D, Jiang S (2015). Evolutionary suppression of erythropoiesis via the modulation of TGF-β signalling in an Antarctic icefish. Mol Ecol.

[44] Haney PJ, Badger JH, Buldak GL, Reich CI, Woese CR, Olsen GJ (1999). Thermal adaptation analyzed by comparison of protein sequences from mesophilic and extremely thermophilic Methanococcus species. Proc Natl Acad Sci U S A.

[45] Mizuguchi K, Sele M, Cubellis MV (2007). Environment specific substitution tables for thermophilic proteins. BMC Bioinformatics.

[46] McDonald JH (2010). Temperature adaptation at homologous sites in proteins from nine thermophile-Mesophile species pairs. Genome Biol Evol.

[47] Suyama M, Torrents D, Bork P (2006). PAL2NAL: robust conversion of protein sequence alignments into the corresponding codon alignments. Nucleic Acids Res.

[48] Peden JF: Analysis of Codon Usage. PhD thesis. Nottingham University; 1999.

[49] Kawashima S, Pokarowski P, Pokarowska M, Kolinski A, Katayama T, Kanehisa M (2008). AAindex: amino acid index database, progress report 2008. Nucleic Acids Res.

[50] Jones DT (1999). Protein secondary structure prediction based on position-specific scoring matrices. J Mol Biol.

[51] Jahandideh S, Abdolmaleki P, Jahandideh M, Barzegari Asadabadi E (2007). Sequence and structural parameters enhancing adaptation of proteins to low temperatures. J Theor Biol.

[52] Madden PW, Babcock MJ, Vayda ME, Cashon RE (2004). Structural and kinetic characterization of myoglobins from eurythermal and stenothermal fish species. Comp Biochem Physiol B Biochem Mol Biol.

[53] Windisch HS, Kathover R, Portner HO, Frickenhaus S, Lucassen M (2011). Thermal acclimation in Antarctic fish: transcriptomic profiling of metabolic pathways. Am J Physiol Regul Integr Comp Physiol.

