# Supplementary material for: GC bias lead to increased small amino acids and random coils of proteins in cold-water fishes
Source: BMC Genomics. 2018 May 2;19:315. doi: 10.1186/s12864-018-4684-z (PMC5930961; doi:10.1186/s12864-018-4684-z)
Supplement: Supplementary file 2 — Table S2. Summary of the fishes investigated in this study. (DOCX 14 kb) [file 12864_2018_4684_MOESM2_ESM.docx]

**Additional file 2: Table S2: Summary of the fishes investigated in this study.**

| Academic name | Abbreviation | Latitude | Average data size (bases)* |
| --- | --- | --- | --- |
| *Gadus morhua* | Gmorh | 80°N - 35°N | 16,232,599 |
| *Gasterosteus aculeatus* | Gacul | 71°N - 26°N | 20,131,405 |
| *Dissostichus mawsoni* | Dmaws | 45°S - 78°S | 12,866,321 |
| *Gymnodraco acuticeps* | Gacut | 61°S - 78°S | 10,896,430 |
| *Oreochromis niloticus* | Onilo | 32°N - 10°N | 19,627,460 |
| *Xiphophorus maculatus* | Xmacu | 23°N - 17°N | 23,450,889 |
| *Danio rerio* | Dreri | 33°N - 8°N | 17,048,406 |

*For each species, the average data size is calculated by averaging the total numbers of nucleotides in pairwise alignments for all species pairs that the species is involved.
